# Spatial ecology and microhabitat selection of the nocturnal pitviper *Viridovipera stejnegeri* (Squamata: Viperidae) in relation to prey

**DOI:** 10.1002/ece3.11445

**Published:** 2024-05-22

**Authors:** Song‐Wen Tan, Ya‐Yong Wu, Jia‐Jun Wang, Bing Lyu, Min Yu, He Zhang, Peng Guo, Lei Shi

**Affiliations:** ^1^ Xinjiang Key Laboratory for Ecological Adaptation and Evolution of Extreme Environment Biology, College of Life Sciences Xinjiang Agricultural University Urumqi China; ^2^ Faculty of Agriculture, Forestry and Food Engineering Yibin University Yibin China

**Keywords:** habitat selection, predation, snake, spatial interaction, *Viridovipera stejnegeri*

## Abstract

Habitat is fundamental for facilitating various life activities in animals, for instance, snakes procure essential energy for survival and reproduction by selecting ambush microhabitats. While there has been extensive research on the selection of microhabitat for feeding in terrestrial and aquatic snakes, little is known about arboreal snakes. In the present study, we analyzed the ambush microhabitat preferences of *Viridovipera stejnegeri*, a widely distributed Asian pitviper in China, conducted association analysis between snake microhabitat and prey microhabitat and abundance to determine the ro5le of microhabitat selection in feeding. Employing random forest analysis and habitat selection functions, we further constructed a predictive framework for assessing the probability of ambush site selection by *V. stejnegeri*. Our results revealed that *V. stejnegeri* exhibited a distinct microhabitat preference for ambush prey. Among the 13 environmental factors assessed, *V. stejnegeri* showed pronounced preferences towards 12 of these factors, including climatic factors, geographical factors, and vegetation factors. Furthermore, although the preferences of *V. stejnegeri* overlapped substantially with those of its prey across multiple habitat factors, food abundance shows no significant association with various habitat factors of *V. stejnegeri*, and does not have significant predictive effect on habitat selection of *V. stejnegeri*. Therefore, we infer that *V. stejnegeri* does not preferentially select microhabitats with the highest food abundance, which does not support the hypothesis that “snakes select habitats based on the spatial distribution of prey abundance.” By analyzing the characteristics of vegetation, geography, and climate, we conclude that *V. stejnegeri* tends to choose microhabitats with better ambush conditions to increase attack success rate, thereby achieving the optimal feeding success rate at the microhabitat scale, which is in line with the predictions of optimal foraging theory. This study provides new insights into the predation ecology and habitat selection of snakes.

## Graphical abstract

In this study, we analyzed the predation microhabitat preferences of *Viridovipera stejnegeri*, a widely distributed Asian pitviper in China, and the role of microhabitat selection in predation. Our results revealed that *V. stejnegeri* exhibited a distinct preference for predation microhabitats. Furthermore, the preferences of *V. stejnegeri* overlapped substantially with those of its prey across multiple habitat factors, support the hypothesis that similar habitat preferences drive spatial interactions.

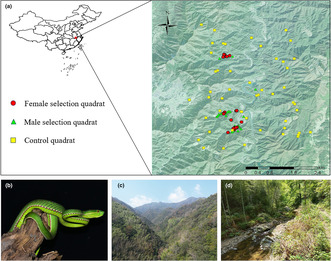

## 1 INTRODUCTION

The suitable microhabitat selection is paramount for wildlife in order to find the essential resources for energy (prey), reproduction (mates), and protection (shelter) (O'Hanlon et al., 2015; Zhang, 2020). Consequently, research on habitat selection has become a prominent topic. Recent advancements in technology, such as radio telemetry, have led to a rapid increase in research on snake habitat selection (Northrup et al., 2022), and the scope of habitat selection research has broadened to encompass various fields, including spatial ecology and distribution prediction (Barnes et al., 2017, 2023; Bartoszek et al., 2021; Natusch et al., 2022; Silva et al., 2020; Strine et al., 2018). These studies are instrumental for unveiling species life histories and animal conservation (Robson & Blouin‐Demers, 2021; Zhang et al., 2020).

Foraging is a critical behavior for acquiring energy and essential for growth and reproduction (Higham et al., 2017). According to the optimal foraging theory, predators are expected to forage in locations that yield the highest success rates (Charnov, 1976). Thus, there is a hypothesis regarding microhabitat selected by snakes to ambush prey, which suggests that snakes select habitats based on the spatial distribution of prey abundance (Tutterow et al., 2021). This has been validated in some snakes, such as the timber rattlesnake (*Crotalus horridus*), which selects ambush habitats based on “overall prey availability” to maximize predation success (Tutterow et al., 2021). However, some researches indicated that at the microhabitat scale, snakes may not necessarily choose the microhabitats with the highest feeding success rates due to factors such as thermoregulation (Blouin‐Demers & Weatherhead, 2001; Carfagno et al., 2006). Thus, additional investigations are needed to determine the applicability of this hypothesis to snake habitat selection at the microhabitat scale. In addition, nocturnal and arboreal species remain understudied due to their elusive nature and habitat, posing challenges for data collection (Fraga et al., 2013).

The nocturnal arboreal Stejneger's Bamboo pitviper (*Viridovipera stejnegeri*) is widely distributed in southern China and Vietnam (Guo et al., 2022; Zhao, 2006). Prior research on the habitat selection of *V. stejnegeri* has been limited to vegetation utilization (Tu et al., 2000), with little attention given to specific habitat or microhabitat preferences for ambush prey. As a nocturnal ambush predator, *V. stejnegeri* can serve as a good model to explore ambush site selection preferences in nocturnal ambush snakes and the role such preferences play in successful predation. Based on previous field observations, we noted that *V. stejnegeri* typically ambushes prey near mountain streams, which is also where its primary prey species, including *Odorrana* and *Amolops* frogs, are distributed. In line with the optimal foraging theory and “snakes select habitats based on the spatial distribution of prey abundance” hypothesis, we predict that *V. stejnegeri* exhibits a clear preference for specific microhabitat features in mountain streams for predation purposes. The preference is related to prey abundance and is advantageous for *V. stejnegeri* to achieve the highest feeding success rate. In the present study, we conducted a detailed analysis of the ambush microhabitat preferences of *V. stejnegeri*, validating the above hypothesis. We also employed Random Forest analysis and habitat selection functions to predict the probability of habitat selection by *V. stejnegeri*. This study will contribute new perspectives to the understanding of predation ecology and habitat selection of snakes.

## 2 MATERIALS AND METHODS

### 2.1 Study sites

**FIGURE 1:**
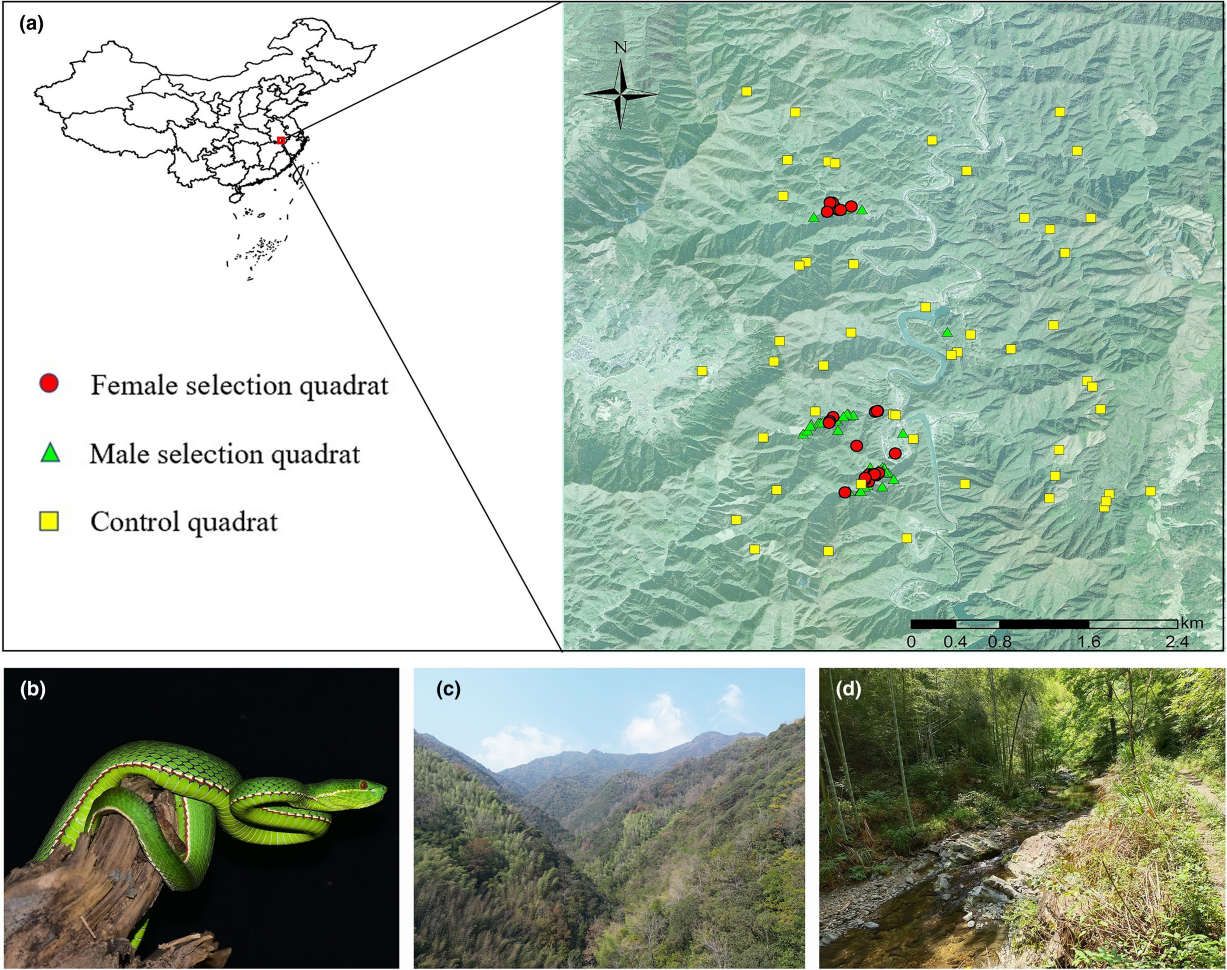
Map showing quadrats of *Viridovipera stejnegeri* (a), general view of study species *V. stejnegeri* (b), landscape habitat of *V. stejnegeri* (c), and microhabitat of *V. stejnegeri* (d).

Data collection was conducted in Huangshan City, Anhui Province, China (117°23′‐118°55′ E, 29°24′‐30°24′ N; Figure 1a). This region is located in eastern China and is characterized by a subtropical monsoon climate with abundant heat and moisture. The annual average temperature ranges from 15.5 to 16.4°C, and average annual precipitation ranges from 1395 to 1702 mm, predominantly falling from May to August. The vegetation is primarily composed of subtropical evergreen broad‐leaved forests, which harbor rich biodiversity (The People's Government of Huangshan City, 2023).

### 2.2 Data acquisition and variable division

To assess the microhabitat preferences of *V. stejnegeri*, we conducted a survey in June 2022, one of the months with the highest precipitation levels, with snake sampling carried out from 20:00 to 24:00 each night within the study area. Independent electronic tags were injected into each located snake, and their unique identification numbers, latitude and longitude coordinates, and sex were recorded. A 4 × 4 m quadrat was established at the sampling location of each snake. Within these quadrats, 13 habitat factors were measured, including altitude, temperature, humidity, landscape habitat, vegetation type, vegetation coverage, vegetation height, slope, aspect, slope position, distance from roads, distance from water (DW), distance from residential sites (DR), and food abundance. Additionally, two ambush parameters, ambush substrate and ambush height, were recorded. Detailed descriptions and measurement methods of various habitat factors are listed in Appendix 1. Due to the limited population density of *V. stejnegeri* and based on previous observations that these snakes alter their ambush sites after either a second ambush event or interference, recaptures were carried out at 7‐day intervals. Independent electronic tags were employed as identification markers for the snakes during these recaptures (Sprague & Bateman, 2018). To minimize the effect of resampling, only those sites located more than 10 m from an individual's previous location were included in microhabitat analysis (Zhang, 2020). Microhabitat data for both *Odorrana* and *Amolops* species within the study area were also collected using the same approach as for the pitviper in June 2023 (except for food abundance).

We also established 60 random quadrats within the survey area using ArcGIS v10.8. Ten quadrats which were located on roads, open water bodies, cliffs, and points within 50 m of *V. stejnegeri* habitats were excluded for being unsuitable habitat for *V. stejnegeri* or preventing issues of false duplication. The remaining 50 quadrats were used as control quadrats, and the aforementioned habitat factors were measured (Zhang, 2020).

### 2.3 Data processing and analysis

A total of 117 quadrats pertaining to the ambush site selection of *V. stejnegeri* were collected, including 90 males and 27 females (with 22 repeated collections. Appendix 2). In addition, 50 control quadrats and 30 quadrats on amphibian habitat selection were also collected (Figure 1a).

**FIGURE 2:**
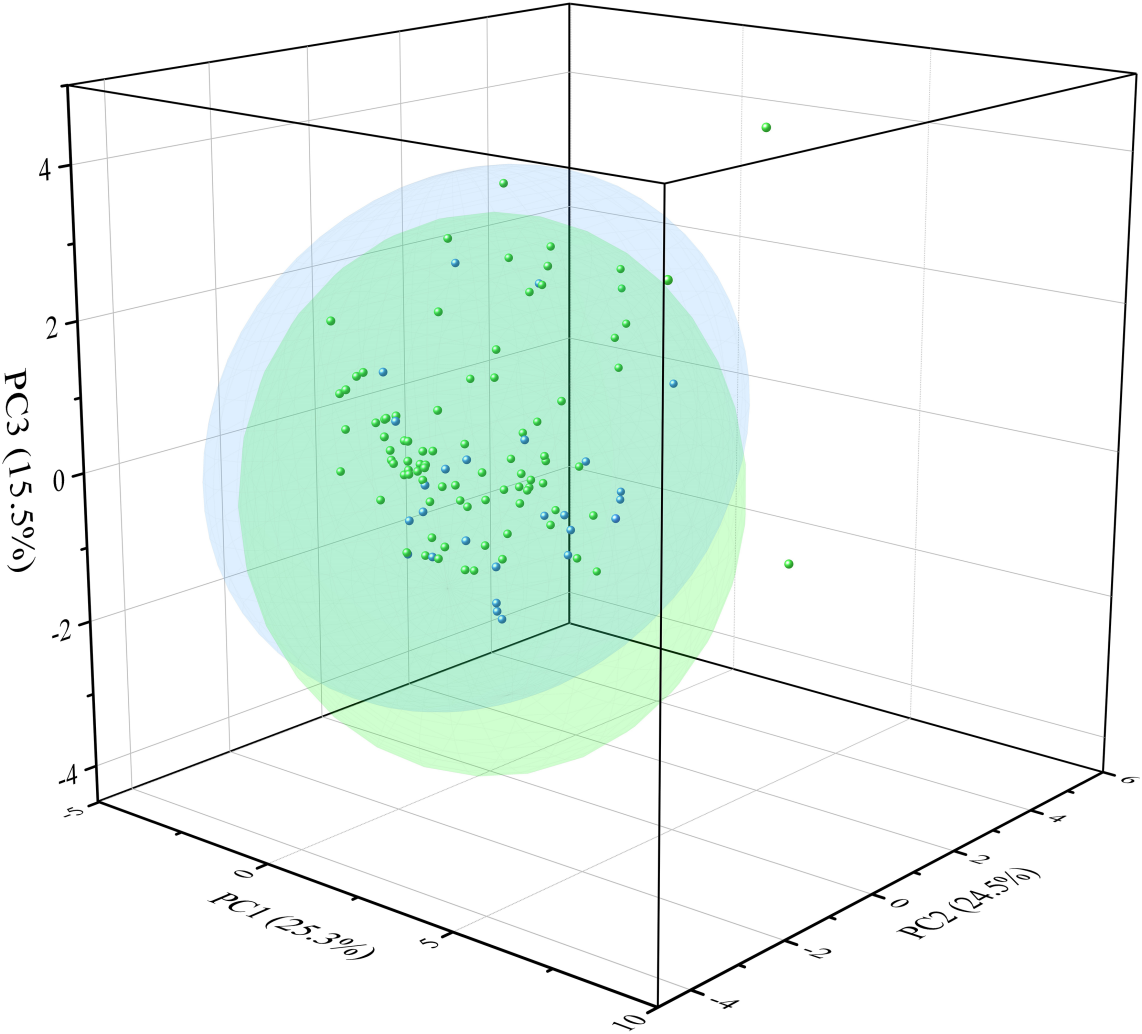
Principal component distribution plot of *Viridovipera stejnegeri*. Green represents males and blue represents females.

Prior to data analysis, normality and homogeneity of variance tests were conducted for all continuous variables. The data were then grouped by sex, and chi‐square tests, Mann–Whitney *U*‐tests, and principal component analysis were conducted, which indicated no significant differences in habitat factors and ambush parameters between males and females overall (Appendix 3, Figure 2). Subsequent analyses were performed by combining males and females. Mann–Whitney *U* and chi‐square tests were used to determine the significance of differences in habitat factors between selection and control quadrats to identify effective habitat factors for further analysis.

We calculated the Vanderploeg (*W*_i_) and Scavia (*E*_i_) Selection Index for each habitat factor to analyze the selection preference of *V. stejnegeri* for each habitat factor (Vanderploeg & Scavia, 1979):

$$W_{i}=\left(\frac{r_{i}}{p_{i}}\right)/\sum \left(\frac{r_{i}}{p_{i}}\right)$$

$$E_{i}=\left(W_{i}-\frac{1}{n}\right)/\left(W_{i}+\frac{1}{n}\right)$$

where *r*_*i*_ denotes the number of quadrats selected by *V. stejnegeri* with characteristics *i*; *p*_*i*_ denotes the total number of quadrats in the environment with characteristics *i*; and *n* refers to the level or category number of a particular habitat factor. *E*_*i*_ ranges from −1 to 1, with *E*_*i*_ = 1 indicating strong preference, 0.1 < *E*_*i*_ < 1 indicating moderate preference, −0.1 < *E*_*i*_ < 0.1 indicating random selection, −1 < *E*_*i*_ < −0.1 indicating low avoidance, and *E*_*i*_ = −1 indicating total avoidance. Furthermore, we assessed the selection preference of *V. stejnegeri* for ambush parameters using the proportion of features appearing in selection quadrats.

To determine potentially similar habitat selection between predator and prey, chi‐square and Mann–Whitney *U*‐tests were performed to assess differences in microhabitat factors between *V. stejnegeri* and *Odorrana* and *Amolops* species. To examine the correlation between habitat selection and food abundance of *V. stejnegeri*, we used Pearson correlation coefficient and Spearman correlation coefficient to test the correlation between continuous variables of various habitat factors and food abundance. We also used Mann–Whitney *U*‐tests to analyze the association between discrete variables of various habitat factors and food abundance.

To predict their distribution and habitat selection, autocorrelation analysis of the continuous variables of *V. stejnegeri* was conducted using Pearson correlation analysis, with highly autocorrelated variables excluded (Gardiner et al., 2015). Random forest analysis was then constructed using continuous variables in the habitat factors of *V. stejnegeri* (R Development Core Team, 2018; Zhang et al., 2020), and the importance of predictive variables in the random forest analysis was plotted according to the mean decrease Gini index. The top five most important habitat variables were selected, and a total of 31 linear logarithmic models incorporating multiple independent habitat variables were built by combining each variable using a generalized linear model (GLM):

$$\text{Logit}\frac{P\left(Y=1\right)}{1-P\left(Y=1\right)}=\beta_{0}+\beta_{1}x_{1}+\beta_{2}x_{2}+\beta_{3}x_{3}+\ldots +\beta_{k}x_{k},$$

where *x* represents different habitat variables and *β* represents selection coefficients.

All models were evaluated based on the Akaike information criterion (AIC; Burnham & Anderson, 2002), with the model showing the smallest ΔAICc value selected as the optimal model. According to the optimal linear model, the probability of *V. stejnegeri* habitat selection (i.e., habitat selection function; Fieberg et al., 2021) is given by:

$$P=e^{\beta_{0}+\beta_{1}x_{1}+\beta_{2}x_{2}+\beta_{3}x_{3}+\ldots +\beta_{k}x_{k}}/\left(1+e^{\beta_{0}+\beta_{1}x_{1}+\beta_{2}x_{2}+\beta_{3}x_{3}+\ldots +\beta_{k}x_{k}}\right)$$

To assess the predictive accuracy of the habitat selection function, its predicted values for the occurrence of *V. stejnegeri* were used as a test variable, with the actual occurrence status serving as the state variable. A receiver operating characteristic (ROC) curve was plotted to evaluate the predictive ability of the model using the area under the curve (AUC; Chen et al., 2007). Additionally, partial dependence plots were used to graphically depict the relationship between each habitat factor and predicted probability of *V. stejnegeri* occurrence obtained from random forest analysis (Zhang, 2020).

All analyses were conducted with a confidence level of 0.05. Data analysis was performed using R v4.2.3 and SPSS 27, and graphs were plotted using R v4.2.3 and Origin 2022.

## 3 RESULTS

### 3.1 Differences between selection and control quadrats

**FIGURE 3:**
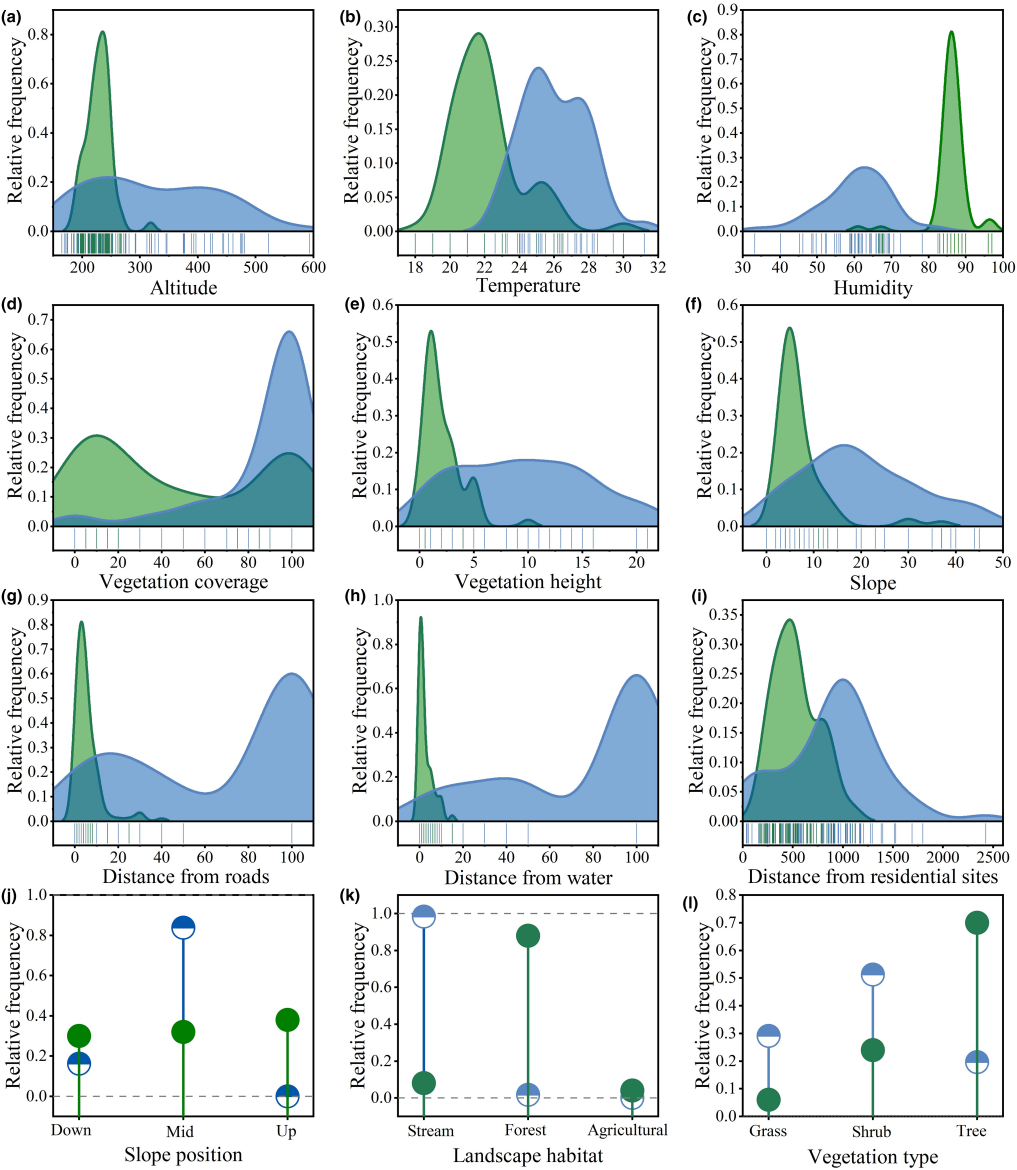
Differences between selection and control quadrats of *Viridovipera stejnegeri*. Only the factors with significant differences were plotted, green represents selection quadrat and blue represents control quadrat. Ordinate (curve height) represents frequency of certain features. (a‐l): frequency of each habitat factor in selection quadrats and control quadrats.

Both Mann–Whitney *U* and chi‐square tests showed significant differences in all variables between the selection and random quadrats (*p* < .05; Appendix 4, Figure 3), with the exception of aspect (*p* = .061). Therefore, aspect was excluded from subsequent analyses.

### 3.2 Ambush microhabitat preference of *V. stejnegeri*

Vanderploeg (*W*_i_) and Scavia (*E*_i_) Selection Index analyses revealed that *V. stejnegeri* exhibited a preference for foraging near grassy areas adjacent to streams, within an elevational range of 200–300 m, with temperatures below 30°C, humidity above 70%, vegetation cover less than 20%, vegetation height less than 5 m, slope less than 15°, located in the mid‐slope position, at distances of less than 10 m from roads, less than 20 m from water, and between 100 and 500 m from residential sites (Appendix 5). Additionally, based on the analysis of ambush parameters, *V. stejnegeri* preferred to ambush prey on rocks near the stream (52%) and typically at an ambush height of less than 20 cm (54%).

### 3.3 Association between *V. stejnegeri* microhabitat with prey microhabitat and abundance

**FIGURE 4:**
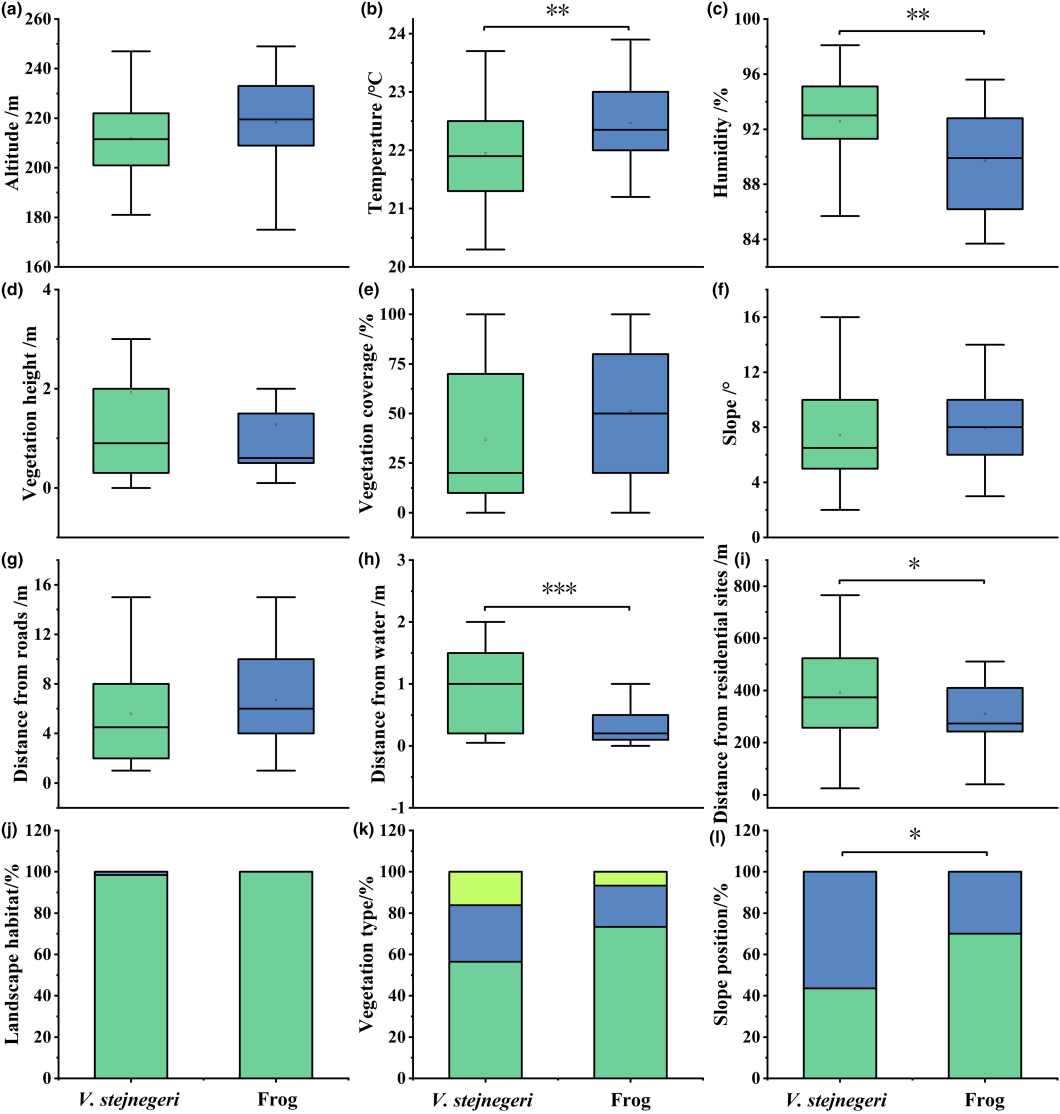
Habitat factor differences between *Viridovipera stejnegeri* and prey. * Significant difference (*p* < .05), ** Highly significant difference (*p* < .01), *** Extremely significant difference (*p* < .001). (a‐l): differences of each habitat factor between *Viridovipera stejnegeri* and frog.

Habitat similarity analysis identified significant differences between *V. stejnegeri* and frogs in terms of temperature (*p* = .005), humidity (*p* = .003), DW (*p* < .001), DR (*p* = .027), and slope position (*p* = .017). However, no significant differences were observed in terms of altitude (*p* = .069), vegetation cover (*p* = .064), vegetation height (*p* = .618), slope (*p* = .238), distance from roads (*p* = .082), landscape habitat (*p* = .484), and vegetation type (*p* = .251) (Figure 4).

The correlation analysis results show that there is no significant correlation between food abundance and various continuous variables. The results of the Mann–Whitney *U*‐test also indicate that there is no significant difference in food abundance among various groups of discrete variables, indicating that the distribution of food abundance is not significantly associated with the discrete variables as well (Appendix 6).

### 3.4 Predicting probability of ambush site selection by *V. stejnegeri*

#### 3.4.1 Random forest model

**FIGURE 5:**
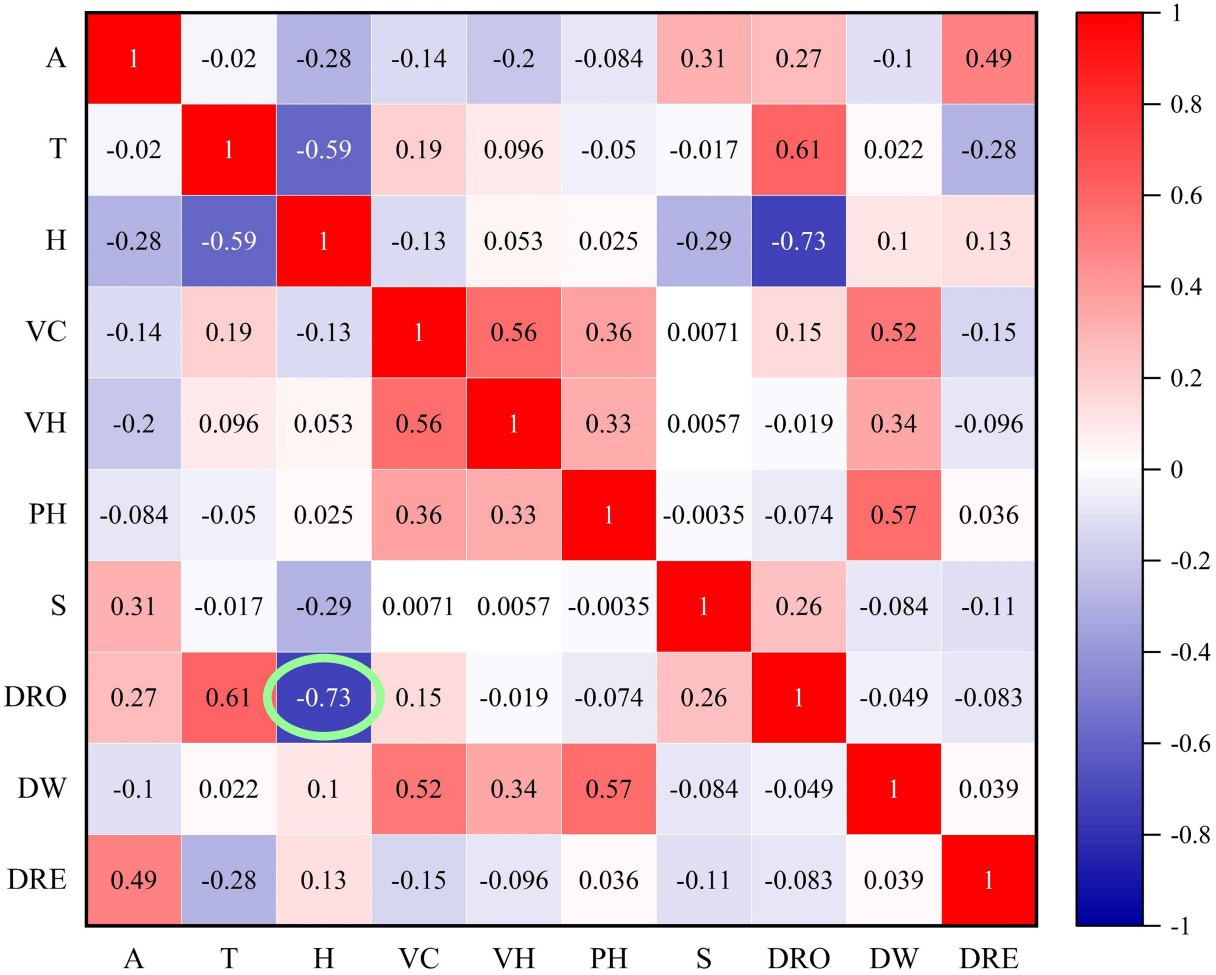
Autocorrelation of habitat factors for prey capture by *Viridovipera stejnegeri*. A: altitude, T: temperature, H: humidity, VC: vegetation coverage, VH: vegetation height, PH: ambush height, S: slope, DRO: distance from roads, DW: distance from water, DRE: distance from residential sites.

**FIGURE 6:**
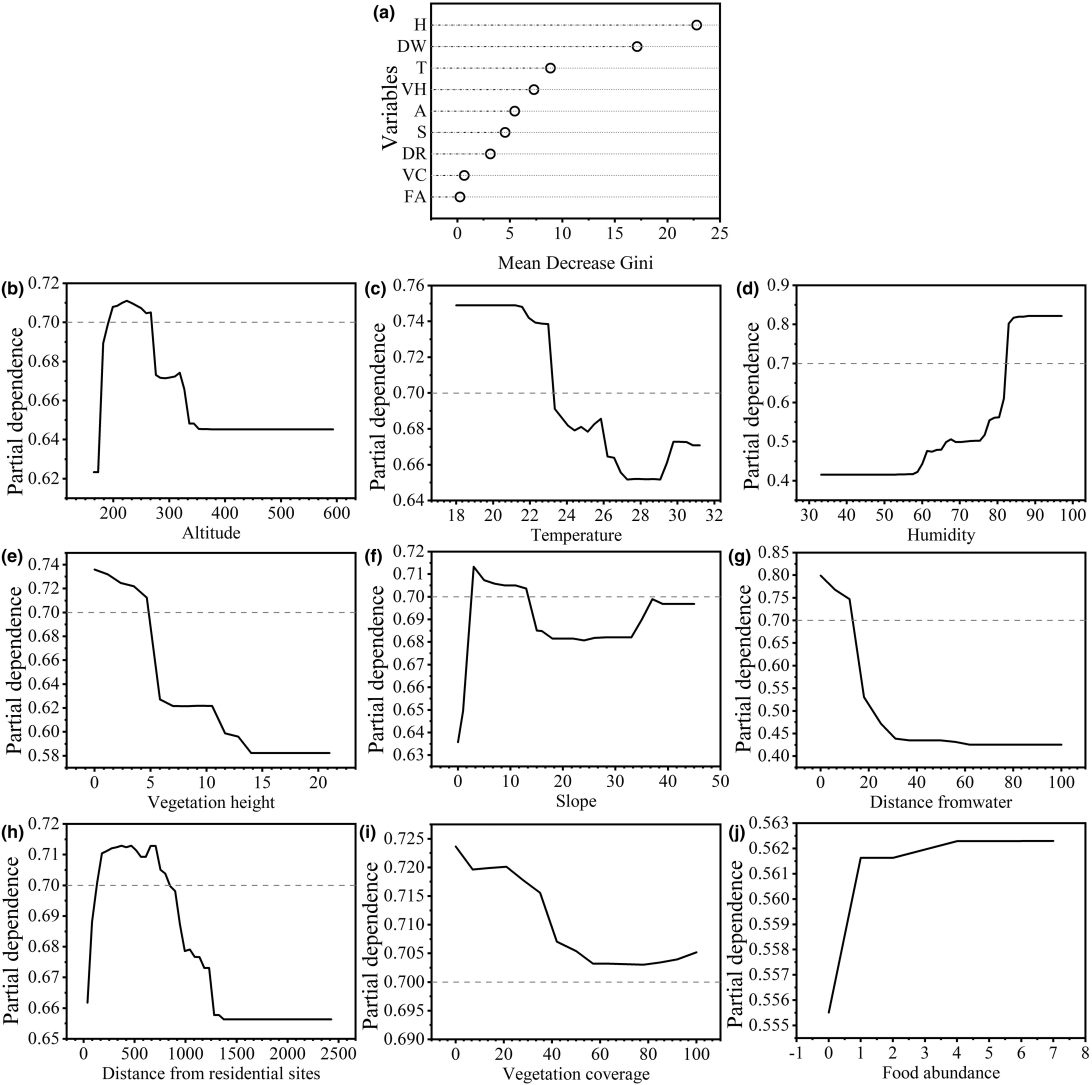
Importance values and partial dependence plots for each predictor variable. (a): importance values for each predictor variable. (b–j): y‐axes are partial dependance. Partial dependance is the dependance of the probability of occurrence on one predictor variable after averaging out the effects of the other predictor variables in the model. A: altitude, T: temperature, H: humidity, VC: vegetation coverage, VH: vegetation height, S: slope, DW: distance from water, DR: distance from residential sites, FA: food abundance.

Autocorrelation analysis demonstrated a significant negative correlation between distance from roads and humidity (*R* = −.73, Figure 5). Consequently, distance from roads was excluded from subsequent analyses. The importance values of variables in the random forest analysis for predicting ambush site selection of *V. stejnegeri*, as determined by the mean decrease Gini index, are shown in Figure 6a. Results revealed that the most important factor influencing selection was humidity (H), followed by DW, temperature (T), vegetation height (VH), altitude (A), slope (S), DR, vegetation coverage (VC), and food abundance (FD). Based on the partial dependence plots from the random forest analysis (Figure 6), the probability of *V. stejnegeri* occurrence was high in habitats with humidity greater than 82%, DW less than 17 m, temperature lower than 23.2°C, VH less than 5 m, altitudes between 190 and 270 m, slopes from 2 to 13°, and DR sites between 100 and 850 m.

#### 3.4.2 Habitat selection function

Among the 31 models constructed using a combination of variables—humidity (H), DW, temperature (T), vegetation height (VH), and altitude (A)—six were identified as overfitted and excluded from further analysis. Thus, 25 models were retained for evaluation. Of these, two models exhibited ΔAICc <2 (Appendix 7). The model with the minimum AIC value identified humidity, DW, and altitude as the most significant predictors for microhabitat selection of *V. stejnegeri*. The associated logistic regression equation is as follows:

$$\text{Logit}\frac{P\left(Y=1\right)}{1-P\left(Y=1\right)}=-21.317+0.238x_{1}-0.331x_{2}-0.032x_{3}$$

In the equation, *x*_1_ represents humidity, *x*_2_ represents DW, and *x*_3_ represents altitude. Consequently, the predictive model for the probability (*p*) of ambush site selection by *V. stejnegeri* is as follows:

$$P=e^{-21.317+0.238x_{1}-0.331x_{2}-0.032x_{3}}/\left(1+e^{-21.317+0.238x_{1}-0.331x_{2}-0.032x_{3}}\right)$$

**FIGURE 7:**
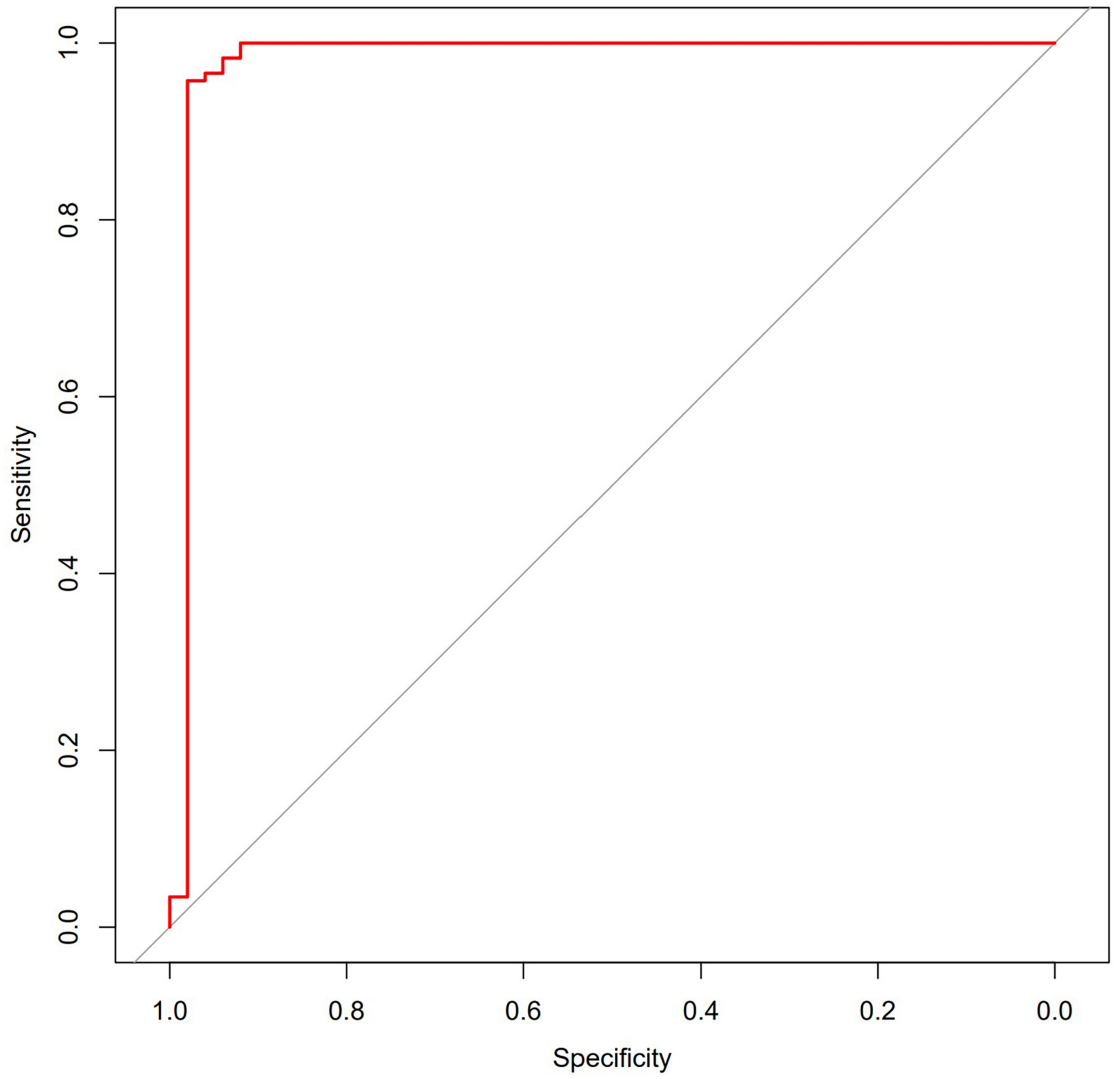
ROC of habitat selection function for *Viridovipera stejnegeri*. AUC, area between red curve and gray diagonal line.

The ROC curve indicated that the predictive ability of this model was excellent (AUC = 0.979; Figure 7).

## 4 DISCUSSION

The optimal foraging theory posits that snakes typically select areas with the highest food abundance for foraging activities to maximize feeding success rate. This study revealed that of the 12 habitat factors for which *V. stejnegeri* showed a preference, seven demonstrated spatial overlap with prey. However, in terms of food abundance, there was no significant correlation between microhabitat factors and food abundance for *V. stejnegeri*. Additionally, the prediction results of the random forest model also showed that food abundance does not have significant predictive effect on habitat selection of *V. stejnegeri*. Based on partial dependence plots, the probability of *V. stejnegeri* occurrence is extremely low when food abundance is less than 0, while the occurrence probability remains relatively consistent when food abundance is greater than 0. Therefore, at the microhabitat scale, *V. stejnegeri* does not strictly adhere to food abundance for microhabitat selection, which does not conform to the hypothesis that “snakes select habitats based on the spatial distribution of prey abundance.”

From a vegetation perspective, *V. stejnegeri* and its prey prefer areas with lower VC and shorter VH near the stream. For frogs, this is mainly due to the need for water and shelter, leading frogs to choose vegetative features that are correlated with water availability, such as southern toads (*Anaxyrus terrestris*; Haggerty et al., 2019; Hinderer et al., 2020). For snakes, low‐growing herbaceous plants is advantageous for foraging. Based on indoor experiments, Shen (2019) advocated that the lower the ambush height of *V. Stejnegeri*, the higher the initial attack success rate. In addition, low vegetation is advantageous for snakes to perceive and attack prey (Padrón et al., 2023; Shen, 2019). Thus, the preference of *V. stejnegeri* for vegetation characteristics not only enhanced the probability of encountering prey through spatial overlap but also presumably improved its attack success rate.

From a geographic perspective, *V. stejnegeri* exhibits differences from its prey in terms of DW, DR sites, and slope position. The reasons for these differences may possibly be attributed to physiological variations between *V. stejnegeri* and its prey (such as DW) or differences in shelter requirements (such as DR sites), as the rock piles along the roads offer *V. stejnegeri* a suitable shelter. The steep terrain of the uphill position and anthropogenic disturbances in the downhill position are potential reasons for the differences in slope position. In summary, geographic features may possibly be one of the main reasons limiting select ambush site with high prey abundance. The complex terrain conditions may lead *V. stejnegeri* to prioritize microhabitats with a high ambush success rate to compensate for the lack of food abundance.

From a climate perspective, there are differences in temperature and humidity preference between *V. stejnegeri* and prey. These distinctions may possibly be closely related to the physiological characteristics and requirements of *V. stejnegeri*, most notably regarding temperature. Both excessively high and low environmental temperatures can restrict the normal activities of snakes (Huey, 1991). These advantages are crucial for metabolic maintenance as well as predation efficiency, suggesting that the temperature preferences of *V. stejnegeri* may have implications beyond predation behavior. The differences in humidity preference may originate from physiological dissimilarities between *V. stejnegeri* and its prey. We conclude that *V. stejnegeri* selects habitats with high humidity and proximity to water to obtain prey. However, due to physiological constraints, *V. stejnegeri* is unable to choose humidity and DW that align with the preferences of its prey. Overall, due to physiological characteristics, climate factors also limit habitat selection of *V. stejnegeri* based on food abundance.

In conclusion, at the microhabitat scale, although vegetation factors do not influence its simultaneous search for high prey abundance and suitable ambush sites, limitations imposed by geographical features and physiological traits prevent *V. stejnegeri* from selecting microhabitats with the highest food abundance. Thus, it tends to select habitats with better ambush conditions to increase attack success rates, thereby achieving the optimal feeding success rate at the microhabitat scale. This aligns with predictions from optimal foraging theory. Indeed, the phenomenon of snakes not choosing microhabitats with high food abundance has been observed in multiple species (Carfagno et al., 2006; Glaudas & Rodríguez‐Robles, 2011; Michael et al., 2014; Sperry & Weatherhead, 2009). In these studies, researchers attribute the reasons for snakes' inability to select habitats based on food abundance mainly to the avoiding behavior of their prey (Glaudas & Rodríguez‐Robles, 2011). Therefore, it is necessary to conduct related studies on the avoiding behavior of amphibians in response to snakes in the future.

One limitation of our study is that due to the impact of the COVID‐19 pandemic, our research lacked longer‐term monitoring, preventing us from determining whether seasonal and annual changes would alter the habitat preferences of *V. stejnegeri*. Our survey was conducted during the rainy season, which may increase the probability of *V. stejnegeri* foraging as amphibians become more active. Additionally, interannual variations in rainfall also have an impact. Therefore, in future studies, we aim to conduct longer monitoring periods to further validate these findings.

## AUTHOR CONTRIBUTIONS

**Song‐Wen Tan:** Conceptualization (lead); data curation (lead); formal analysis (supporting); methodology (equal); validation (equal); visualization (supporting); writing – original draft (equal); writing – review and editing (equal). **Ya‐Yong Wu:** Conceptualization (supporting); data curation (supporting); formal analysis (lead); methodology (equal); software (lead); validation (equal); writing – review and editing (equal). **Jia‐Jun Wang:** Conceptualization (supporting); data curation (supporting); formal analysis (lead); investigation (equal). **Bing Lyu:** Conceptualization (supporting); data curation (supporting); validation (equal); writing – review and editing (equal). **Min Yu:** Formal analysis (supporting); investigation (supporting); writing – review and editing (equal). **He Zhang:** Conceptualization (supporting); data curation (supporting); software (supporting). **Peng Guo:** Conceptualization (lead); data curation (lead); formal analysis (supporting); investigation (supporting); methodology (supporting); software (supporting); visualization (supporting); writing – original draft (equal); writing – review and editing (supporting). **Lei Shi:** Formal analysis (supporting); investigation (supporting); methodology (lead); software (supporting); visualization (supporting); writing – original draft (equal); writing – review and editing (supporting).

## CONFLICT OF INTEREST STATEMENT

The authors declare no conflicts of interest.

## Supporting information


Appendix 1.

Appendix 2.

Appendix 3.

Appendix 4.

Appendix 5.

Appendix 6.

Appendix 7.

## Data Availability

All raw data and analysis code are stored in Dryad (DOI: 10.5061/dryad.83bk3jb11). In addition, we provide a “Private for Peer Review” link: https://datadryad.org/stash/share/675_FkUOoPg1afuDI_fOTZtQ1VowQs3V7UwjEHFDPpg. All analyses were performed with publicly available programs. All analysis codes can be publicly accessed and utilized.
