# Supplementary material for: Spatial ecology and microhabitat selection of the nocturnal pitviper Viridovipera stejnegeri (Squamata: Viperidae) in relation to prey
Source: Ecol Evol. 2024 May 22;14(5):e11445. doi: 10.1002/ece3.11445 (PMC11109613; doi:10.1002/ece3.11445)
Supplement: Supplementary file 1 — Appendix 1. [file ECE3-14-e11445-s005.docx]

**Appendix 1 Description and division of habitat factors and predation parameters**

| Variable | Description | Division |
| --- | --- | --- |
| Altitude (m) | Altitude in quadrat was directly measured by GPS. | 1. < 200 m; 2. 200–300 m; 3. > 300 m. |
| Temperature (℃) | Ambient temperature in quadrat was measured using a portable hand-held electronic temperature and hygrometer. During the measurement process, the thermohygrometer is placed at the location of the snakes. Once the values have stabilized, readings are taken and recorded. This procedure is repeated three times consecutively, and the average value is calculated. | 1. < 20 ℃; 2. 20–30 ℃; 3. > 30 ℃. |
| Humidity (%) | Ambient humidity in quadrat was measured by a portable hand-held electronic temperature and hygrometer. The measurement method is analogous to temperature measurement. | 1. < 40%; 2. 40%–70%; 3. > 70%. |
| Landscape habitat | Habitat types within 300 m of the quadrat were assessed. | 1. Stream; 2. Forest; 3. Agricultural. |
| Vegetation type | Main vegetation types in quadrat were assessed. | 1. grass; 2. Shrub; 3. Tree. |
| Vegetation height (m) | Vegetation height of herbs and shrubs was measured by tape measure, arbor height was calculated by trigonometric function. | 1. 0–2 m; 2. 2–5 m; 3. > 5 m. |
| Vegetation coverage (%) | When the sun shone directly at noon during the day, long (a) and short diameters (b) of shadows were measured with a tape measure, then calculated using the formula 1/4πab. | 1. < 20%; 2. 20%-70%; 3. > 70%. |
| Slope (°) | Average slope of quadrat was measured using a handheld GPS. | 1. 0°-15°; 2. 15°-40°; 3. > 40°. |
| Slope position | Slope position of quadrat was estimated. | 1. Downhill position; 2. Mid-slope position; 3. Uphill position. |
| Aspect (°) | Quadrat aspect was recorded with GPS. | 1. Sunny slope (135©alf-shaded and half-sunny slope (45°-135°，225°-315°). |
| Distance from roads (m) | Distance of quadrat from the road (m) was measured using a tape measure. | 1. 0 m-10 m; 2. 10 m-30 m; 3. > 30 m. |
| Distance from water (m) | Distance of quadrat from water was measured using a tape measure. | 1. < 5 m; 2. 5 m-20 m; 3. > 20 m. |
| Distance from residential sites (m) | Distance of quadrat from residential sites was measured using the ArcGIS v10.8 Mapping tool. | 1. < 100 m; 2. 100 m-500 m; 3. > 500 m. |
| Ambush substrate | Ambush substrate of *V. stejnegeri* was recorded, only in selected quadrat. | 1. Rock; 2. Cliff; 3. Vegetation. |
| Ambush height (cm) | Ambush height of *V. stejnegeri* was measured using a tape measure, only in selected quadrat. | 1. < 20 cm; 2. 20 cm-100 cm; 3. > 100 cm. |
| Food abundance | Recording the number of preys within quadrats. | 1. < 2; 2. 2-5; 3. > 5. |
