# Supplementary material for: Spatial ecology and microhabitat selection of the nocturnal pitviper Viridovipera stejnegeri (Squamata: Viperidae) in relation to prey
Source: Ecol Evol. 2024 May 22;14(5):e11445. doi: 10.1002/ece3.11445 (PMC11109613; doi:10.1002/ece3.11445)
Supplement: Supplementary file 2 — Appendix 2. [file ECE3-14-e11445-s007.docx]

**Appendix 2 Information of effective recaptures**

| ID | Date of initial arrest | Date of recapture | Interval time (d) | Spacing distance (m) |
| --- | --- | --- | --- | --- |
| 2510 | June 5th | June 12th | 7 | 15 |
| 2470 | June 5th | June 12th | 7 | 18 |
| 2550 | June 5th | June 12th | 7 | 16 |
| 2504 | June 5th | June 19th | 14 | 52 |
| 2479 | June 6th | June 13th | 7 | 18 |
| 2486 | June 6th | June 13th | 7 | 12 |
| 2459 | June 6th | June 20th | 14 | 22 |
| 2498 | June 7th | June 21st | 14 | 27 |
| 2465 | June 7th | June 28th | 21 | 16 |
| 2549 | June 7th | June 14th | 7 | 11 |
| 2548 | June 7th | June 14th | 7 | 18 |
| 2473 | June 7th | June 14th | 7 | 17 |
| 2527 | June 12th | June 19th | 7 | 13 |
| 2454 | June 12th | June 26th | 14 | 20 |
| 2521 | June 13th | June 20th | 7 | 15 |
| 2524 | June 13th | June 20th | 7 | 15 |
| 2509 | June 13th | June 27th | 14 | 11 |
| 2460 | June 14th | June 28th | 14 | 19 |
| 2462 | June 14th | June 21st | 7 | 31 |
| 2508 | June 19th | June 26th | 7 | 16 |
| 2464 | June 19th | June 26th | 7 | 22 |
| 2518 | June 21st | June 28th | 7 | 13 |
