# Supplementary material for: Spatial ecology and microhabitat selection of the nocturnal pitviper Viridovipera stejnegeri (Squamata: Viperidae) in relation to prey
Source: Ecol Evol. 2024 May 22;14(5):e11445. doi: 10.1002/ece3.11445 (PMC11109613; doi:10.1002/ece3.11445)
Supplement: Supplementary file 3 — Appendix 3. [file ECE3-14-e11445-s002.docx]

**Appendix 3 Ambush site selection differences between males and females of *V. stejnegeri***

| Variable | Males  (Mean ± SD)  (*n* = 90) | Females  (Mean ± SD)  (*n* = 27) | Mann-Whitney U test | chi-square test |
| --- | --- | --- | --- | --- |
| Altitude (m) | 231.30 ± 22.16 | 220.00 ± 18.69 | ***P* = 0.031** |  |
| Temperature (℃) | 21.81 ± 1.94 | 23.00 ± 2.09 | ***P* = 0.004** |  |
| Humidity (%) | 85.77 ± 5.34 | 86.22 ± 2.79 | *P* = 0.298 |  |
| Vegetation coverage (%) | 48.11 ± 40.87 | 45.37 ± 40.64 | *P* = 0.756 |  |
| Vegetation height (m) | 2.01 ± 1.73 | 2.26 ± 2.05 | *P* = 0.524 |  |
| Ambush height (m) | 42.11 ± 82.20 | 43.70 ± 81.77 | *P* = 0.750 |  |
| Slope (°) | 7.21 ± 6.41 | 7.26 ± 5.45 | *P* = 0.519 |  |
| Aspect (°) | 137.91±95.54 | 140.13±97.51 | *P* = 0.132 |  |
| Distance from water (m) | 2.47 ± 3.09 | 2.85 ± 4.19 | *P* = 0.361 |  |
| Distance from roads (m) | 5.54 ± 6.64 | 5.96 ± 5.96 | *P* = 0.424 |  |
| Distance from residential sites (m) | 560.16 ± 234.32 | 440.67 ± 197.76 | ***P* = 0.009** |  |
| Landscape habitat |  |  |  | *P* = 0.610 |
| Vegetation type |  |  |  | *P* = 0.887 |
| Slope position |  |  |  | *P* = 0.199 |
| Ambush substrate |  |  |  | *P* = 0.052 |
