# Supplementary material for: Spatial ecology and microhabitat selection of the nocturnal pitviper Viridovipera stejnegeri (Squamata: Viperidae) in relation to prey
Source: Ecol Evol. 2024 May 22;14(5):e11445. doi: 10.1002/ece3.11445 (PMC11109613; doi:10.1002/ece3.11445)
Supplement: Supplementary file 4 — Appendix 4. [file ECE3-14-e11445-s003.docx]

**Appendix 4 Differences between selection and control quadrats of *V. stejnegeri***

| Variable | Selection quadrat  (Mean ± SD)  (*n* = 117) | Control quadrat  (Mean ± SD)  (*n* = 50) | Mann-Whitney U test | Chi-square test |
| --- | --- | --- | --- | --- |
| Altitude (m) | 228.69 ± 21.86 | 322.96 ± 110.57 | *P <* 0.001 |  |
| Temperature (℃) | 22.09 ± 2.03 | 26.01 ± 1.85 | *P* < 0.001 |  |
| Humidity (%) | 85.87 ± 4.87 | 60.55 ± 9.16 | *P* < 0.001 |  |
| Vegetation coverage (%) | 47.48 ± 40.66 | 87.20 ± 24.83 | *P* < 0.001 |  |
| Vegetation height (m) | 2.06 ± 1.80 | 9.54 ± 5.94 | *P* < 0.001 |  |
| Slope (°) | 7.22 ± 6.18 | 19.72 ± 12.13 | *P* < 0.001 |  |
| Aspect (°) | 137.01 ± 95.91 | 174.68 ± 101.76 | *P* = 0.061 |  |
| Distance from water (m) | 2.56 ± 3.36 | 76.04 ± 35.30 | *P* < 0.001 |  |
| Distance from roads (m) | 5.64 ± 6.47 | 68.46 ± 40.41 | *P* < 0.001 |  |
| Distance from residential sites (m) | 532.58 ± 231.19 | 904.6 ± 488.50 | *P* < 0.001 |  |
| Landscape habitat |  |  |  | *P <* 0.001 |
| Vegetation type |  |  |  | *P <* 0.001 |
| Slope position |  |  |  | *P* < 0.001 |
