# Supplementary material for: Spatial ecology and microhabitat selection of the nocturnal pitviper Viridovipera stejnegeri (Squamata: Viperidae) in relation to prey
Source: Ecol Evol. 2024 May 22;14(5):e11445. doi: 10.1002/ece3.11445 (PMC11109613; doi:10.1002/ece3.11445)
Supplement: Supplementary file 5 — Appendix 5. [file ECE3-14-e11445-s001.docx]

**Appendix 5 Selective analyses of habitat factors of *V. stejnegeri***

| Variable | Category | Expected proportion used (*p_i_*) | Proportion used (*r_i_*) | Resource selection coefficient (*W_i_*) | Resource selection index (*E_i_*) | Preference |
| --- | --- | --- | --- | --- | --- | --- |
| Altitude (m) | < 200 | 21 | 12 | 0.36 | 0.04 | RS |
|  | 200–300 | 119 | 113 | 0.60 | 0.28 | MP |
|  | > 300 | 27 | 2 | 0.05 | -0.76 | LA |
| Temperature (℃) | < 20 | 3 | 3 | 0.59 | 0.28 | MP |
|  | 20–30 | 163 | 114 | 0.41 | 0.11 | MP |
|  | > 30 | 1 | 0 | 0.00 | -1.00 | TA |
| Humidity (%) | < 40 | 1 | 0 | 0.00 | -1.00 | TA |
|  | 40–70 | 49 | 4 | 0.08 | -0.62 | LA |
|  | > 70 | 117 | 113 | 0.92 | 0.47 | MP |
| Landscape habitat | Stream | 119 | 115 | 0.96 | 0.48 | MP |
|  | Forest | 46 | 2 | 0.04 | -0.77 | LA |
|  | Agricultural | 2 | 0 | 0.00 | -1.00 | TA |
| Vegetation type | Herb | 37 | 34 | 0.43 | 0.12 | MP |
|  | Shrub | 72 | 60 | 0.39 | 0.08 | RS |
|  | Arbor | 58 | 23 | 0.18 | -0.29 | LA |
| Vegetation coverage (%) | < 20 | 49 | 47 | 0.42 | 0.12 | MP |
|  | 20–70 | 36 | 29 | 0.36 | 0.03 | RS |
|  | > 70 | 82 | 41 | 0.22 | -0.20 | LA |
| Vegetation height (m) | < 2 | 65 | 62 | 0.52 | 0.22 | MP |
|  | 2–5 | 65 | 53 | 0.45 | 0.15 | MP |
|  | > 5 | 37 | 2 | 0.03 | -0.84 | LA |
| Slope (°) | < 15 | 123 | 109 | 0.82 | 0.42 | MP |
|  | 15–40 | 41 | 8 | 0.18 | -0.30 | LA |
|  | > 40 | 3 | 0 | 0.00 | -1.00 | TA |
| Slope position | Down | 34 | 19 | 0.39 | 0.08 | RS |
|  | Mid | 114 | 98 | 0.61 | 0.29 | MP |
|  | Up | 19 | 0 | 0.00 | -1.00 | TA |
| Distance from roads (m) | < 10 | 100 | 95 | 0.58 | 0.27 | MP |
|  | 10–30 | 32 | 20 | 0.38 | 0.07 | RS |
|  | > 30 | 35 | 2 | 0.04 | -0.81 | LA |
| Distance from water (m) | < 5 | 89 | 88 | 0.54 | 0.24 | MP |
|  | 5–20 | 35 | 29 | 0.46 | 0.16 | MP |
|  | > 20 | 43 | 0 | 0.00 | -1.00 | TA |
| Distance from residential sites (m) | < 100 | 6 | 1 | 0.10 | -0.54 | LA |
|  | 100–500 | 60 | 55 | 0.54 | 0.24 | MP |
|  | > 500 | 101 | 61 | 0.36 | 0.04 | RS |

Note: LA represents low avoidance; TA represents total avoidance; RS represents random selection; MP represents moderate preference.
