# Supplementary material for: Spatial ecology and microhabitat selection of the nocturnal pitviper Viridovipera stejnegeri (Squamata: Viperidae) in relation to prey
Source: Ecol Evol. 2024 May 22;14(5):e11445. doi: 10.1002/ece3.11445 (PMC11109613; doi:10.1002/ece3.11445)
Supplement: Supplementary file 6 — Appendix 6. [file ECE3-14-e11445-s006.docx]

**Appendix 6 Association between microhabitat selection and food abundance of *V. stejnegeri***

| Variables | Relativity analysis (*R*) | Mann-Whitney U test (*P*) |
| --- | --- | --- |
| Altitude (m) | 0.174 |  |
| Temperature (℃) | -0.010 |  |
| Humidity (%) | -0.090 |  |
| Vegetation coverage (%) | -0.075 |  |
| Vegetation height (m) | -0.080 |  |
| Slope (°) | -0.024 |  |
| Distance from water (m) | 0.123 |  |
| Distance from roads (m) | 0.037 |  |
| Distance from residential sites (m) | 0.198 |  |
| Landscape habitat |  | 0.708 |
| Vegetation type |  | 0.385 |
| Slope position |  | 0.606 |
