# Supplementary material for: Spatial ecology and microhabitat selection of the nocturnal pitviper Viridovipera stejnegeri (Squamata: Viperidae) in relation to prey
Source: Ecol Evol. 2024 May 22;14(5):e11445. doi: 10.1002/ece3.11445 (PMC11109613; doi:10.1002/ece3.11445)
Supplement: Supplementary file 7 — Appendix 7. [file ECE3-14-e11445-s004.docx]

**Appendix 6 Evaluation results of top 10 models for ambush site selection by *V. stejnegeri***

| Model ID | Models | *k* | AICc | ΔAICc | Weight |
| --- | --- | --- | --- | --- | --- |
| 1 | H + DW + A | 5 | 25.12 | 0.00 | 0.44 |
| 2 | H + DW + VH + A | 6 | 26.20 | 1.09 | 0.26 |
| 3 | H + DW + VH | 5 | 27.70 | 2.58 | 0.12 |
| 4 | H + DW | 4 | 28.37 | 3.25 | 0.09 |
| 5 | H + DW + T + VH | 6 | 29.63 | 4.51 | 0.05 |
| 6 | H + DW + T | 5 | 30.04 | 4.93 | 0.04 |
| 7 | DW + T + VH | 5 | 34.89 | 9.77 | < 0.01 |
| 8 | DW + T + VH + A | 6 | 36.30 | 11.18 | < 0.01 |
| 9 | DW + T | 4 | 37.15 | 12.03 | < 0.01 |
| 10 | H + VH | 4 | 37.26 | 12.14 | < 0.01 |

Note: Models were ranked according to AIC.
